# Electrospun Polylactide—Poly(ε-Caprolactone) Fibers: Structure Characterization and Segmental Dynamic Response

**DOI:** 10.3390/polym16101307

**Published:** 2024-05-07

**Authors:** Svetlana G. Karpova, Anatoly A. Olkhov, Ivetta A. Varyan, Oksana I. Khan, Andrey A. Botin, Anna V. Naletova, Anatoly A. Popov, Alexey L. Iordanskii

**Affiliations:** 1 Department of Biological and Chemical Physics of Polymers, Emanuel Institute of Biochemical Physics, Russian Academy of Sciences, 4 Kosygina Street, 119334 Moscow, Russia; karpova@sky.chph.ras.ru (S.G.K.); aolkhov72@yandex.ru (A.A.O.); anatoly.popov@mail.ru (A.A.P.); 2 Academic Department of Innovational Materials and Technologies Chemistry, Plekhanov Russian University of Economics, 36 Stremyanny Lane, 117997 Moscow, Russia; 3 Institute of Biochemical Technology and Nanotechnology, RUDN University, 6 Miklukho-Maklaya Street, 117198 Moscow, Russia; oksa_0096@mail.ru; 4 N. N. Semenov Federal Research Center for Chemical Physics Academy of Science, 119991 Moscow, Russia; 5 Department of Organic Chemistry and Petroleum Chemistry, Gubkin University, 65 Leninsky Prospect Building 1, 119991 Moscow, Russia; botin-andrey@mail.ru (A.A.B.); naletovaann96@gmail.com (A.V.N.)

**Keywords:** polylactide, polycaprolactone, electrospun fibers, EPR correlation time, amorphous phase, crystalline phase

## Abstract

Electrospun ultrathin fibers based on binary compositions of polylactide (PLA) and poly(ε-caprolactone) (PCL) with the various content from the polymer ratio from 0/100 to 100/0 have been explored. Combining thermal (DSC) and spectropy (ESR) techniques, the effect of biopolymer content on the characteristics of the crystal structure of PLA and PCL and the rotative diffusion of the stable TEMPO radical in the intercrystallite areas of PLA/PCL compositions was shown. It was revealed that after PLA and PCL blending, significant changes in the degree of crystallinity of PLA, PCL segment mobility, sorption of the Tempo probe, as well as its activation energy of rotation in the intercrystalline areas of PLA/PCL fibers, were evaluated. The characteristic region of biopolymers’ composition from 50/50 to 30/70% PLA/PCL blend ratio was found, where the inversion transition of PLA from dispersive medium to dispersive phase where an inversion transition is assumed when the continuous medium of the PLA transforms into a discrete phase. The performed studies made it possible, firstly, to carry out a detailed study of the effect of the system component ratio on the structural and dynamic characteristics of the PLA/PCL film material at the molecular level.

## 1. Introduction

Bio-based polyesters are currently being successfully employed in medicine, environmental remediation, and packaging owing to their high biocompatibility, enzymatic degradation, and eco-friendly behavior, without the negative impacts on living organisms and eco-systems [1,2,3,4]. Currently, polylactides (PLA) are getting great attention owing to their important features such as biodegradation, aqueous hydrolysis, commercial availability, and others. The final products of the PLA decomposition are carbon dioxide and water, which are safe for the human population [5,6,7]. Bioresorbable polylactide implants offer advantages for many medical applications such as tissue engineering, bone fixation devices, and as a bioresorbable material in orthopedics and maxillofacial surgery [8,9]. Besides, due to the abovementioned benefits, perfect mechanical and diffusional characteristics, the PLA family is used in various industries, including automotive, computer industry, food packaging industry, electronic appliances, and 3D printing [10,11,12]. However, a number of mechanical disadvantages, e.g., brittleness, are obstacles to the practical application of PLA and its derivatives [13].

Commonly, a successful approach to the softening of many plastics is to add a soft elastomeric component by blending. This traditional approach can also be applied to biodegradable polymer blends. The elastic poly(ε-caprolactone) (PCL) as a softening agent is a biocompatible and biodegradable semi-crystalline polyester with a melting point in the range 55–70 °C [14]. PCL and their copolymers have been shown to be the most commonly used polymers in a wide range of clinical applications: sutures, system drug delivery, coronary stents, fixation screws, etc. This polymer can be biodegraded by external living organisms (bacteria and fungi), but it is not biodegradable in animal and human organisms due to a lack of suitable enzymes [15]. Considering the qualities of all polymers, polycaprolactone polyether (PCL), in addition to being biocompatible and biodegradable, is finding increasing use due to its affordability, cost effectiveness, and suitability for modification. Its regulated physicochemical state, biological properties, and mechanical strength allow it to resist physical, chemical, and mechanical influences without significant loss of its properties [16,17,18]. PCL biodegrades slowly compared to other polymers, so it is most suitable for long-term delivery lasting more than 1 year. PCL also has the ability to form compatible blends with other polymers that can influence degradation kinetics, facilitating adaptation for achieving desired release profiles [19,20].

This work focuses on the study of PLA–PCL blends produced as electrospun fibers. Two principal advantages are achieved when these components are blended. On the one hand, the proper polyesters’ compositions are more successfully engaged in biomedical applications [21], modern therapy for drug delivery platform design [22], packaging industrial coatings [23], and in many other areas. On the other hand, a series of the drawbacks inherent to individual blend constituents (PLA and PCL) can be successfully eliminated by the blend fabrication. For example, without special processing, pristine PLA is characterized by poor mechanical features, slow crystallization, low thermal resistance, and other rates of degradation [24]. Analogously, pristine PCL reveals low mechanical strength, slow degradation rate that prevents the implementation of polyester [25].

Nowadays, there are a significant number of publications that focus on the exploration of PLA–PCL blends as biodegradable synergetic systems with improved viscoelastic, thermal, mechanical, and barrier characteristics [17,26,27]. An essentially lower number of papers were devoted to this system’s fabrication in ultrathin fibrillar forms obtained by electrospinning. This non-sophisticated technique opens a new horizon for the production and application of nano- and microfibers in such promising technologies as water treatment, designing 3D scaffolds for tissue engineering, fabrication of smart nonwoven textiles, and electro-writing [28,29,30].

From the literature evidence, it is definitely known that the PLA/PCL blends are mostly immiscible with the formation of separated phases that vary their dimensions from a nano- to a micro-scope [17,18,25,31]. In such a situation, interphase interaction, geometry and structure of the phases, and molecular dynamics (segmental mobility) are dominantly responsible for the tensile and fracture parameters, as well as for permeability, diffusion transport, and drug capacity characteristics. The pioneering paper devoted to the PLA/PCL system exploration has reported Pluronic^TM^ plasticizing for the PLA/PCL blend matrices [17], which was confirmed by the complex of methods such as DSC, SEM, MDA.

One of the main goals of PLA/PCL blends is the development of materials with improved properties while maintaining the biodegradability and biocompatibility of both phases. Unfortunately, the low melting point of PCL limits its use in a wide range of applications, especially at elevated temperatures. However, melting PLA with PCL can reduce the brittleness of the former component, offering a wider range of potential applications. In [32], they found excellent morphology and remarkable improvements in tensile elongation and impact toughness that are interpreted in the framework of the DSC curves, which have shown the effect of PCL content on the glass transition temperature (Tg), melting temperature, and degree of crystallinity of PLA/PCL blends when the melting enthalpies of both components of the blend are decreased sharply [33].

It was shown in [18] that at PCL content up to 20–25 wt%, the blend has a fine phase structure with small particles and a narrow particle-size distribution. At 30 wt%, the total volume of the PCL phase becomes larger, and the particle size distribution belonging to the dispersed areas expands. At a PCL content of more than 40 wt%, the phase morphology becomes continuous, which is followed by a decrease in PLA melting enthalpy from 31 to 24 kJ/mol. It is interesting, that the strength of the 80/20 PLA/PCL blend exceeds the strength of the pure impact-resistant PCL working as the modifier; that reflects rather a synergistic effect of blending. Recently, for the PLA/PCL system, another interesting effect of shape memory was discovered [34,35]. This specific behavior consists of the ability of the binary blend to change its initial geometry when exposed to a specific external stimulus, usually temperature, and to regain its original form reproducibly when the stimulus is stopped.

One of the advanced methods for producing ultrafine fibers is electrostatic forming, or electrospinning forming, of polymer solutions and melts. The main advantages of this nanotechnology include a relatively low cost of equipment, simplicity of tooling, variability of conditions for fiber obtaining, as well as a variety of different types of fibers produced [12]. The use of bio-based polymers, such as PHB, PLA, and PCL, provides additional advantages in the development of fiber and matrix systems for environmental applications and in biomedicine. Fibrillar matrices or mats formed by nanofibers create favorable conditions for free migration and proliferation of cells in the three-dimensional space of framework structures and, accordingly, provide high integration affinity of the material to living tissues [36]. They are actively used in the design of biosensors and nanofilters, for wound therapy and the immobilization of enzymes, for the creation of prolonged and targeted drug delivery platforms, and in other areas of modern biology and medicine [37].

The greatest practical and commercial advantages are found in composite ultrathin fibers [20,38], which have a high specific surface area, structural diversity, and the ability to effectively control drug delivery. Obtaining polymeric modifications of biologically active compounds is an actively developing branch of chemical technology and is aimed at the synthesis of polymeric materials with reasonable biomedical properties.

The careful insight into macromolecular dynamics expressed via segmental mobility is an essential step that is needed for a comprehensive consideration of the key processes promoting structure–morphology evolution during bioplastic exploitation. Such fundamental phenomena as biodegradation, diffusion, melting/crystallization, phase reconstructions, mechanical behavior, and many others are implemented at the molecular and nanoscopic levels through segment movement, which has been comprehensively reviewed by Veksli et al. [39]. Along with widely used methods such as NMR, mechanic or dielectric dynamic spectroscopy, thermophysical execution, and IR (FTIR, ATR, and Raman) spectroscopy, the spin-probing ESR spectroscopy technique presents an extremely responsive approach to the reliable exploration of segmental mobility in petrol- and bio-based polymer blend systems [40,41]. Earlier, the authors investigated a series of individual and blended biopolymers on the base of poly(3-hydroxybutyrate) [PHB] by the above-mentioned ESR method to elucidate the role of segmental dynamics in annealing, hydrothermal aging, ozonolysis, as well as for water and drug diffusion in the PHB-based films and electrospun fibers [42,43,44]. The correlation among thermophysical phenomena, diffusivity, and segmental dynamics has stimulated us to transfer the focus of academic consideration from PHB blends to PLA ones. Nowadays, the latter polymer systems are more in demand in many innovative areas of human activity. It should be noted that for PLA, the crystallinity is initially lower than for PHB. Consequently, for this polyester, as for PCL, the ratio between the amorphous and the crystalline phases increases compared to the similar characteristics of PHB that allow the explorer to accommodate the higher concentration of the probe in the intercrystalline area of the PLA/PCL blend at the same temperature and, hence, to enhance the ESR method sensitivity until 10–11 spin/cm^3^.

Taking into account the above knowledge, the objectives of this paper are to obtain the versatile fibrillar composition based on the biodegradable polymers, PLA and PCL, by electrospinning; firstly, to evaluate the segmental mobility of the polymer molecules in the PLA/PCL blends by the spin probing ESR technique; and to elucidate the relationship between the dynamic parameters characterized by the correlation time of spin-probe rotation and thermal parameters derived from the corresponding DSC curves.

## 2. Materials and Methods

We used bio-based polylactide (PLA) of NatureWorks^®^ Ingeo™ 3801X Injection Grade PLA (SONGHAN Plastics Technology Co., Ltd., Fengxian District, Shanghai City, China) brand with an average viscosity molecular weight of 190 kDa and polyε-caprolactone pellets (Mw~80 kDa, Sigma-Aldrich, St. Louis, MO, United States) polycaprolactone (PCL), melting temperature 60 °C.

Ultra-thin PLA/PCL fibers were produced by electrospinning [45,46,47] on an EFV-1 single-capillary lab scale unit (Moscow, Russia). The capillary diameter is 0.2 mm. The distance between the capillary and the precipitation electrode was 20 cm. The voltage was 17 kV. Feed solutions of PLA/PCL in chloroform were prepared at different polymer ratios. The total concentration of polymers in the solution was 7 wt%. Stirring was carried out using a magnetic stirrer at 50 °C.

Electron paramagnetic resonance (EPR) X-band spectra were recorded on an EPR-V automated spectrometer (Federal Research Center for Chemical Physics, Russian Academy of Sciences, Moscow, Russia). A stable nitroxide radical TEMPO was used as a spin probe. At a temperature between 50 and 70 °C, the radical was introduced into the fibers from the gas phase for an hour. The value of the microwave power to avoid saturation effects did not exceed 1 mW. The modulation amplitude was always much smaller than the resonance line width and did not exceed 0.5 G. The concentration of the radical in the polymer was determined by double integration of the EPR spectra; the reference was an evacuated solution of TEMPO in CCl4 with a radical concentration of ~10^−3^ mol/L. The probe rotation correlation times τ in the region of fast rotations (5 × 10^−11^ < τ < 10^−9^ s) were found from the EPR spectra by the formula [48]:

τ = ΔH^+^ [(I^+^/I^−^)^0.5^ − 1] 6.65 × 10^−10^ (1)

where ΔH^+^—width of the spectrum component located in the weak field, I^+^/I^−^—ratio of intensities of the components in the weak and strong field, respectively. The measurement error τ was ±5%.

The equilibrium concentration of the adsorbed radical in the samples of the studied compositions of the same mass was calculated using Brucker Winer simfonia software. In the process of taking spectra, the amplification was recorded, the sample was weighed, and then the calculation of the radical concentration in each sample was made in the Origin program.

The samples were investigated by the DSC method using the NETSZCH STA 449 F5 Jupiter(New Castle, DE, USA). Briefly, 50–70 mg of the sample was loaded into a corundum crucible and heated in a stream of nitrogen at a flow rate of 100 mL/min in the temperature range from 20 to 250 degrees at a rate of 2 degrees per minute.

The morphology of nonwoven samples was studied using a laser 3D microscope (LEXT OLS 4100, Olympus, Tokyo, Japan). The method consisted of the fact that the sample was fixed on a cover slip and placed on the microscope stage. Then, the area of the surface and the required magnification were selected, the focus was adjusted, and the image was taken. Microphotographs were processed using a standard software package (OLS 4100 version 3.1.5).

## 3. Results

### 3.1. PLA/PCL Fiber Geometry

**Figure 1:**
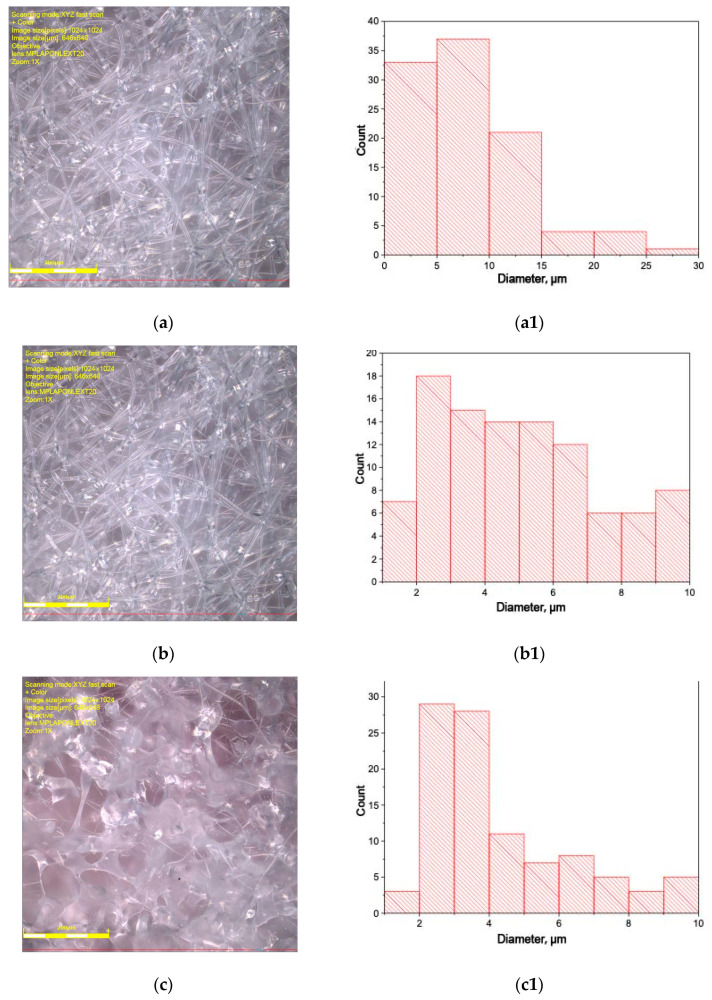

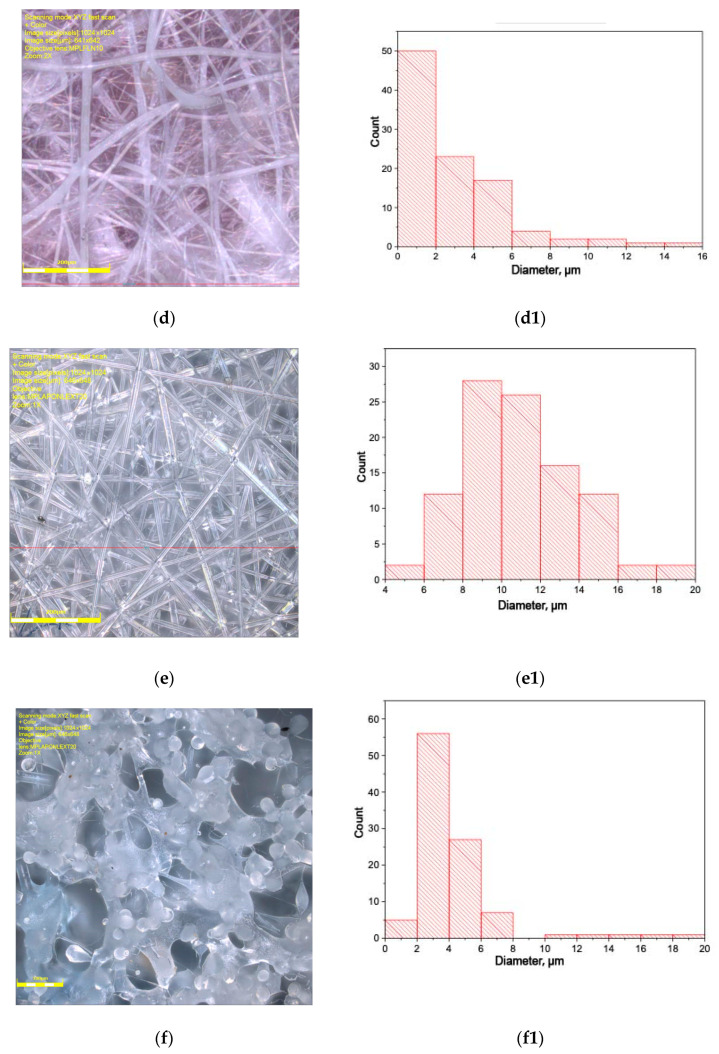

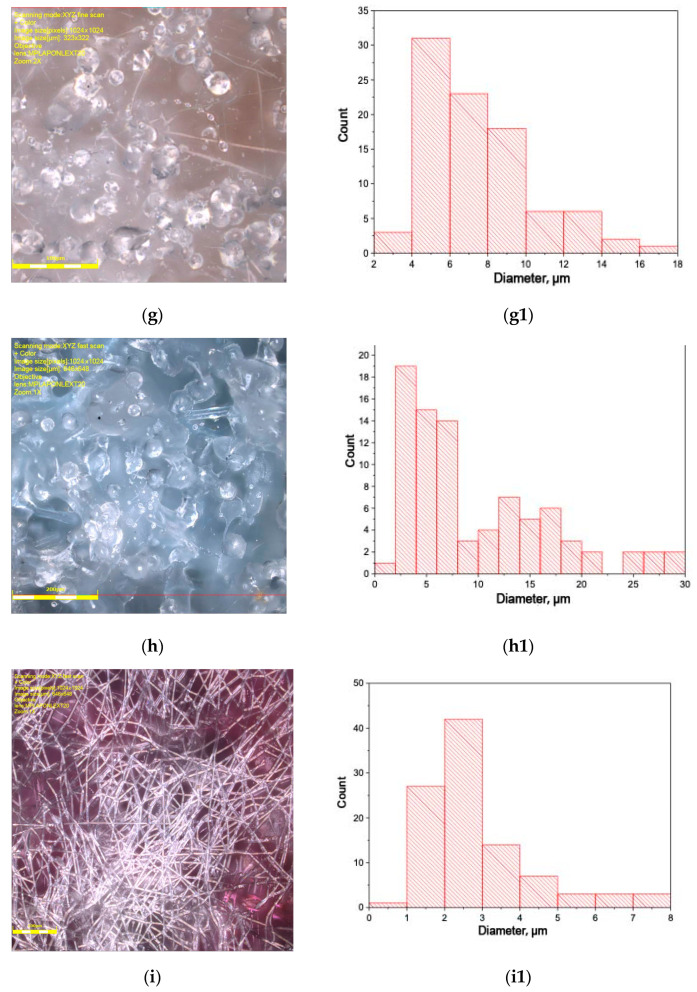
Microphotographs of PLA–PCL fiber materials: 100-0 (**a**); 90-10 (**b**); 80-20 (**c**); 70-30 (**d**); 50-50 (**e**); 30-70 (**f**); 20-80 (**g**); 10-90 (**h**); 0-100 wt% (**i**). The right column shows the corresponding histograms of fiber diameter distribution (**a1**–**i1**).

Figure 1 shows microphotographs of nonwoven fiber materials PLA–PCL. Fibers based on polymer blends have multiple thickenings in the form of beads. The size of the thickenings varies from 10 to 40 µm. The value of error amounted to 5% (Figure 1(a1,c1–g1,i1)) and 3% (Figure 1(b1,h1)).

The cause of thickening is related to the rheological features of PLA and PCL in the blends. The tensile dynamics of polymer solutions seems to be complicated by relaxation processes in polymer phases at the interface and different values of surface tension due to the molecular characteristics of polymers. This is evidenced by the diameter values of individual polymers: PLA fibers have diameters of 5–15 μm and PCL 2–5 μm (Figure 1a,i, respectively). The values of cylindrical fiber diameters in PLA–PCL blends lie in the range of 2–12 μm. The large variation in the values of blend fiber diameters is due to the processes of splitting of the primary jet, the unsteady rheological behavior of binary blends during capillary passage, and the pulling of the solution drop in the field of electrostatic and hydrodynamic forces. We should pay attention to the morphology of fibers based on the PLA–PCL blend: 50–50 wt.%. They have an even, cylindrical shape and practically no thickening. Apparently, it is connected with the formation of structure from two continuous phases, while at other ratios, one of the polymers in the blend acts as the disperse phase, and the formation of a jet tends to coagulation and the formation of drops. It is quite possible that the level of intermolecular interaction between PLA and PCL influences the process of formation of the blended fiber geometry. Based on the analysis of the morphology of blended fibers, we can assume a rather low level of interaction between PLA and PCL. However, to confirm or refute these conclusions, it is necessary to conduct a structural–dynamic analysis of the crystalline and amorphous regions in the blended fibers.

### 3.2. Thermal Characteristics of PLA/PCL Binary Fiber Composition

The processes of biodegradation, oxidation, and hydrolysis affect not only the morphology of the surface of the film or fiber material but also the structure and concentration in the fiber of the amorphous and crystalline phases formed in the volume of the composite [49,50,51]. Polymers in blends have a mutual influence on crystallization processes. Let us consider the influence of the addition of PCL to PLA on the crystalline structure of blended compositions.

In the process of blending two polymers (PLA and PCL) along with the change in morphology of ultrafine fibers, one should expect changes in their thermophysical and structural characteristics. The DSC method was used to study the thermophysical characteristics of the fibers. For this purpose, thermograms of PLA and PCL fibers and their blended compositions were obtained.

**Figure 2:**
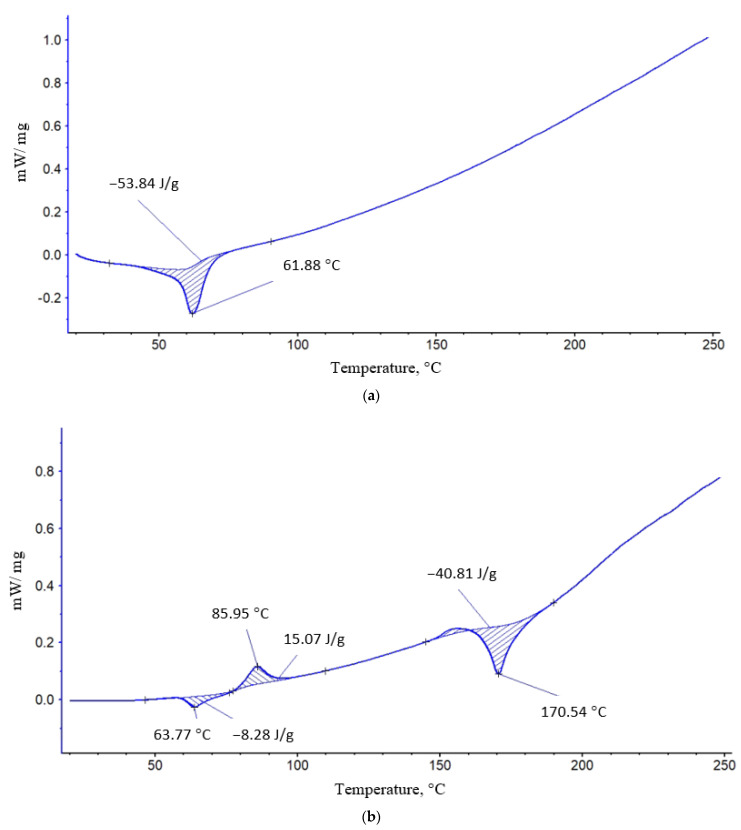

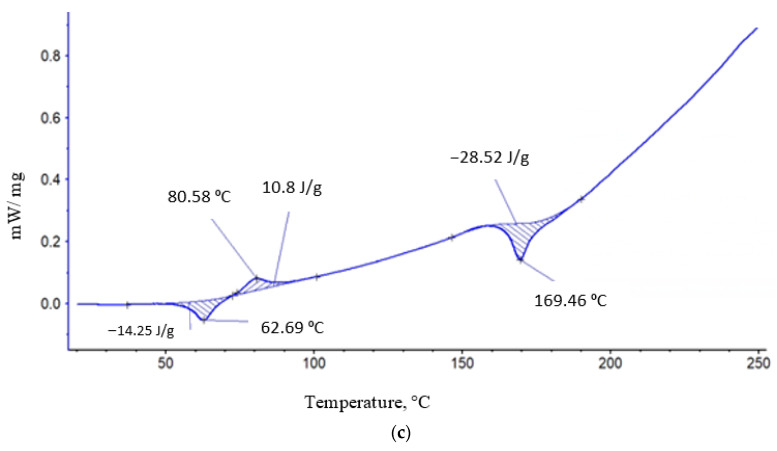
Thermograms of melting of initial PCL (**a**), PLA (**b**), PCL/PLA 50/50 (**c**).

Figure 2 shows the heating peaks of PLA (a) and PCL (b). The DSC curves of polymer samples of different compositions with component ratios ranging from 100/0 to 50/50% PLA/PCL showed 3 characteristic peaks: Two endothermic peaks and one exo-peak. A characteristic feature of these thermograms is the overlapping of the endothermic peaks of the melting of PCL and the glass transition of PLA, which complicates the calculation of the enthalpy of the melting of PCL. The presence of exopeak at 85.95 °C in PLA indicates the process of cold crystallization and formation of linear structures in the fibers, which indicates the presence of a large proportion of straightened chains in the polymer, which are characterized by a large entanglement at temperatures below 85.95 °C. At higher temperatures, there appears to be the possibility of forming crystalline and linear structures.

**Figure 3:**
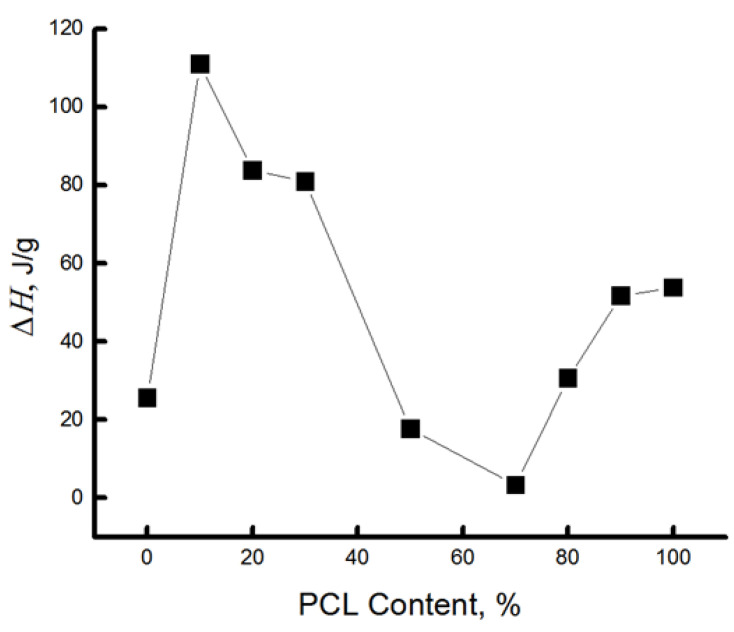
Dependence of ΔH on fiber composition.

With the addition of 10% PCL, the enthalpy of melting PLA increases dramatically (Figure 3) to the highest value. It can be assumed that the first portion of PCL is distributed in the system in the form of tiny particles, thereby plasticizing the structure of PLA, and, as a result, the proportion of straightened chains increases sharply; ΔH increases almost 4 times relative to the initial PCL crystallinity.

At a low concentration of PCL in the system of particles of this polymer, they move in the form of a finely dispersed phase and act as crystallization centers. At the same time, the enthalpy of the cold crystallization of PLA decreases sharply (2-fold). It was shown in [37] that at PCL content up to 20–25 wt %, the blend has a thin phase structure with small particles and a narrow particle-size distribution. At 30 wt. %, the content of the dispersive phase structure enlarges, and the particle size distribution expands. At a PCL content of more than 40 wt %, the morphology of the phases becomes continuous, with the PLA-rich phase partially dissolving the PCL. With increasing concentrations of PCL, ΔH begins to decrease; apparently, the particle size distribution expands, and the effect of plasticization and additional crystallization begins to decrease.

Now let us consider the change in enthalpy of the melting of PCL. The complexity of calculating this parameter consists of superimposing of the PCL melting peak (T_m_ = 62 °C) and the PLA glass transition peak (T_gt_ = 64 °C) on the thermogram. However, the total value of ΔH can be used to estimate the change in the melting enthalpy of PCL when blended with PLA. Table 1 shows that when PCL is added to the blended composition up to and including 70%, its ΔH is extremely low, i.e., the proportion of crystallites and linear systems in PCL is negligible. The cold crystallization of fiber observed in DSC thermograms for PLA and its blends (see results in Table 1) suggests the formation of structural structures with oriented polymer chains at relatively low temperatures, below ~80 °C. The appearance of such a structure corresponds to force fields (viscous and electric), which determine the progress of electrospinning.

As PCL is introduced into the blended composition, its initial ratio between the enthalpies of cold crystallization and melting of PLA (*ΔH*cc/*ΔH*cr), reflecting its crystalline state, decreases markedly, which may be a consequence of the interaction of the components during the fiber preparation process. As a result of the increasing number of PLA–PCL segmental contacts, the spatial orientation of PLA chains is disturbed to some extent, and the contribution of cold crystallization becomes less pronounced.

The results confirm the conclusion about the fine-dispersed distribution of PCL in the PLA matrix when it is added to the blend up to 70%. And only starting from the 30/70% PLA/PCL composition, when polycaprolactone forms a continuous phase, the formation of crystallites and linear systems of PCL occurs in the blend, the proportion of which increases significantly with increasing concentrations of the latter in the blend (Table 1).

Let us point out the reason for the spontaneous straightening and additional orientation of the chains. It is known from thermodynamics that the conformational criterion k* = h/L (where h is the average distance between chain ends and L is the contour chain length) determines the tendency of a macromolecule to straightening, additional orientation, or folding into a coil. Each polymer has its own critical value k*. Chains with k > k* tend to have additional orientation; macromolecules with k < k* tend to take a coil conformation. Thus, during the process of forming a fiber material, a certain part of straightened macromolecules is formed, but the system does not have time to completely transition to an equilibrium state. At elevated temperatures, recrystallization occurs with an increase in crystallite sizes, and the proportion of structures consisting of straightened chains increases. At the same time, the structure of the amorphous regions also changes.

The nature of changes in the thermal characteristics of the binary PLA/PCL system depending on the composition of polymer components allows us to draw a preliminary conclusion that there is a certain region of concentrations between 50/50 and 30/70% of PLA/PCL where phase inversion takes place, i.e., when the continuous (dispersed) phase of PLA transforms into a dispersed phase. In this concentration range, the enthalpy of melting of PLA and PCL is characterized by very low values; there are no exo-peaks characteristic of PLA; and there is a kink in the dependence of ΔH on composition (Figure 3).

The addition of small concentrations of PCL into the system promotes the formation of a certain fraction of more perfect crystallites with a melting point of about 220 °C (the melting range lies between 160 and 220 °C), while in the original PLA this range lies between 160 and 190 °C. It is important to note that the DSC method provides information about both the proportion of three-dimensional crystallites and the proportion of linear structures with two-dimensional order.

With an increase in the concentration of PCL over 70% in the system, the enthalpy of melting of both PLA and PCL increases sharply (Table 1). In the region of compositions with 80–90% PCL content, the enthalpy of melting of this polymer is 33.6 and 33.8 J/g, while in the homopolymer, it is 54 J/g. It can be assumed that at these compositions, interfacial layers are formed, which partially prevent the formation of the crystalline phase.

Thus, the study of the crystalline phase (of also linear systems) of PLA, PCL, and their blended compositions has shown that the addition of polycaprolactone to PLA from 10 to 30% causes a sharp increase in the enthalpy of melting in this composition, and this is due to the plasticizing effect of finely dispersed particles of PCL. In the range of 50–70% PLA/PCL, apparently, there is a phase inversion, which leads to a sharp decrease in the enthalpy of melting. A further increase in the concentration of PCL in the blend is accompanied by a sharp increase in ΔH in both polycaprolactone and PLA. Since the enthalpy of melting in the blended compositions with PCL content ranging from 70 to 90% is significantly lower than in the homopolymer, it can be assumed that interphase interlayers are formed in this material. A characteristic feature of these blended compositions is the decrease in the melting temperature of PLA as the concentration of PCL increases, starting from the composition 50/50% at 170.5 to 165.1 °C.

Changes in the enthalpy of melting of the polymers under study, and therefore the degree of crystallinity, are accompanied by structural changes in the amorphous phase of the system, which will be analyzed further using the ESR method.

### 3.3. Dynamic Characteristics of the Amorphous Phase of Blended PLA/PCL Compositions

For partially crystalline polymers, the structure of the amorphous regions is largely inhomogeneous. Depending on whether the polymers in the composition are compatible or incompatible and how the crystalline component of the mixture changes, the structure of the amorphous phase will also change. As shown in the previous section, the enthalpy of fusion, and therefore the degree of crystallinity, changes dramatically when PCL is added to PLA.

**Figure 4:**
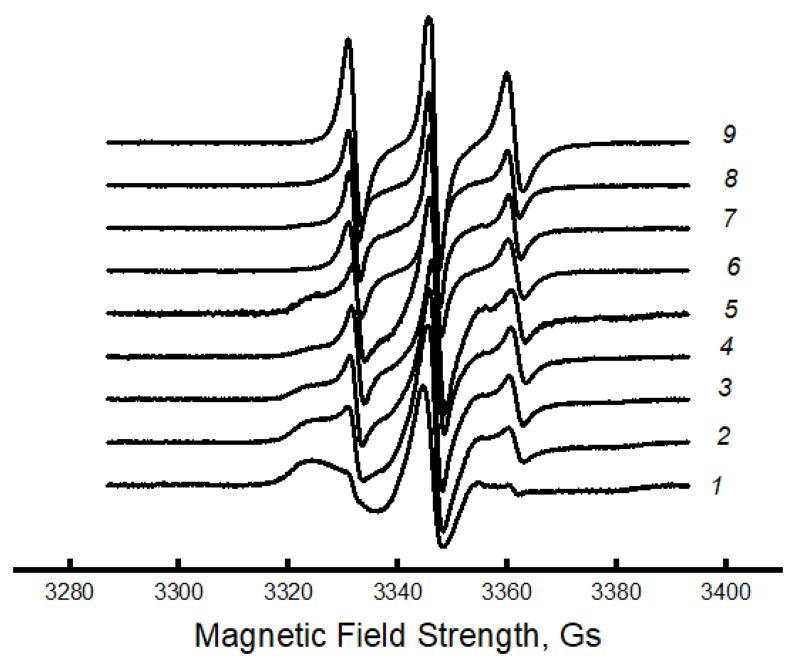
EPR spectra for PLA/PCL fibers: *1*—100/0, *2*—90/10, *3*—80/70, *4*—70/30, *5*—50/50%, *6*—70/30%, *7*—80/20, *8*—90/10, *9*—0/100%.

Consequently, one can expect dramatic changes in the structure of amorphous regions. To study molecular mobility, we applied the EPR technique using the stable nitroxide radical TEMPO, which acts as a molecular probe. Let us consider the effect of the composition of the PLA/PCL mixture on the dynamics of polymer molecules. It was previously shown that the EPR spectra of a radical in a PLA matrix are a superposition of two spectra corresponding to two radical populations with their characteristic correlation times τ1 and τ2 [48,52,53]. The correlation time τ1 reflects the mobility of molecules in denser amorphous regions (slow component of the spectrum), and τ2 reflects mobility in less dense regions (fast component of the same spectrum). The spectra of these polymers are shown in Figure 4.

**Figure 5:**
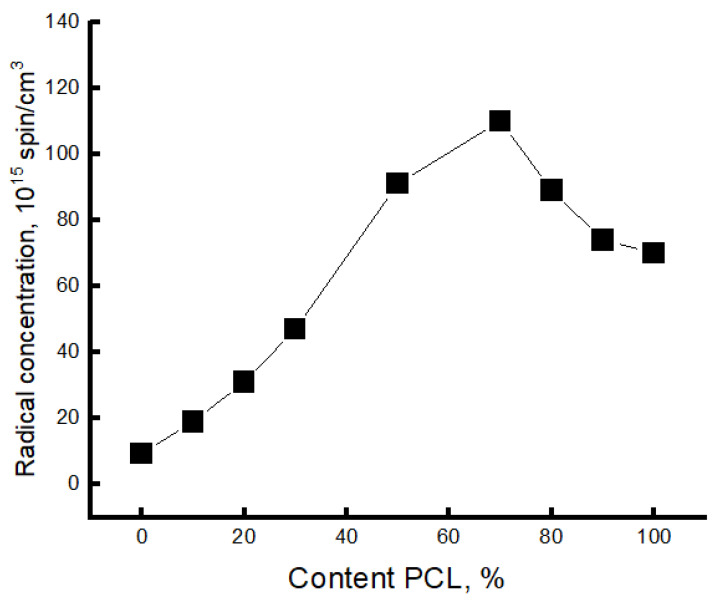
Changes in radical concentration from the composition of the PLA/PCL blend.

It can be seen that the spectra of PLA samples and PLA/PCL blended compositions with PLA content up to 70% look like a two-component system, i.e., there is an overlap of two spectra with slow and fast components. For samples with a PCL content of 70% or more, the spectrum is a single component. Amorphous regions of polymers most often have a heterogeneous nature, which is due to the difference in the packing density of polymer molecules. Structures of straightened chains are characterized by fairly dense packing and, accordingly, low molecular mobility. Macromolecules with a folded conformation form structures with a lower packing density and, accordingly, with higher molecular mobility. Because of their high potential barrier for internal rotation of chain links, PLA molecules are very rigid. The glassy dense mesh of this polymer prevents effective penetration of the radical, as confirmed by its low concentration in the samples (the radical was introduced at 50 °C). Using a special Simfonia and Winer program, concentrations in different density areas were evaluated. PCL has an elastic amorphous phase with high sorption properties. The amorphous phase of PLA is characterized by significantly higher correlation times and low radical concentrations. As the PCL content in the blend increases, the radical concentration increases dramatically (as shown in Figure 5).

It is in the region of 50–70% of the PCL content that a kink is observed in this dependence. As it was already shown in the previous section, the PCL concentration range of 50–70% belongs to the phase inversion range, where practically all characteristics change their character depending on the composition, and a phase transition takes place when the dispersed phase of the PLA transforms into the dispersed phase.

**Figure 6:**
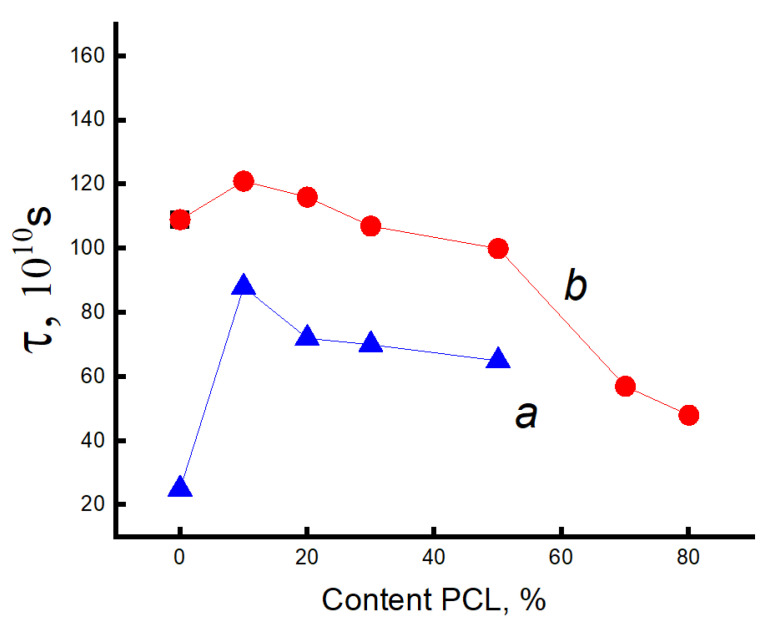
Dependence of τ1 on the content of the composition when introducing the radical into the system at 50 °C (**a**) and at 70 °C (**b**).

Using mathematical processing of the experimental spectra using a special program, Simfonia and Winer (Bruker^®^, Krailing, Germany), the correlation times in PLA and PCL with different proportions in the blend were calculated. Subtracting the spectrum of PCL from the spectra of the blend composition, the correlation time τ1 in PLA was calculated (Figure 6a).

**Figure 7:**
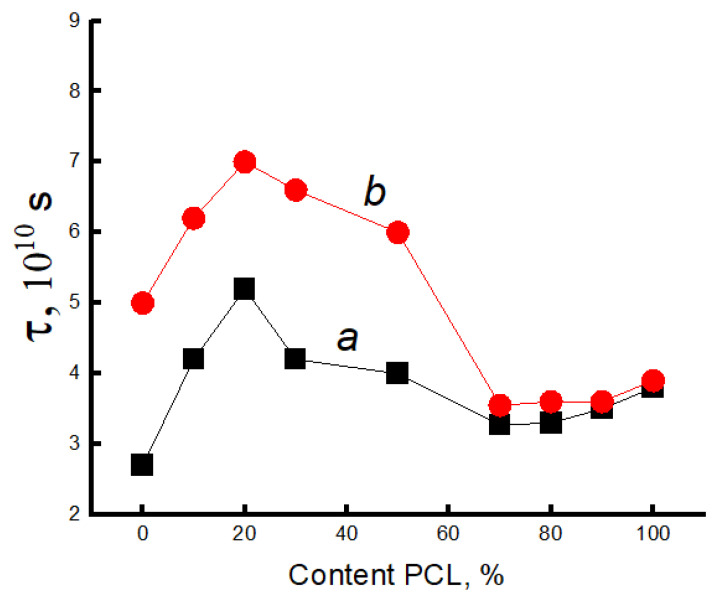
Dependence of τ2 on the content of the composition when introducing the radical into the system at 50 °C (**a**) and at 70 °C (**b**).

The correlation time τ2 was calculated similarly (Figure 7a).

The Figure 7 shows that this parameter increases sharply (τ1 and τ2) when up to 20% PCL is introduced into the system, which suggests the presence of a transition of the amorphous structure of PLA from a glassy to a highly elastic state. The addition of up to 20% PCL causes its finely dispersed distribution in the system (as shown in [37]), which plasticizes the structure of the blend and causes the growth of straightened macromolecules in the composition, and, as a result, the molecular dynamics increases dramatically. In the concentration range from 20 to 50% PCL, a decrease in τ1, τ2 is observed, which is caused by a decrease in the effect of plasticization of the structure by PCL particles due to an increase in their size [37]. At higher polycaprolactone concentrations in the blend (from 50 to 70%), a kink is observed in the dependence of τ2 on the polymer composition, and further growth of the PCL concentration in the system leads to weak changes in the correlation time, despite a significant increase in the enthalpy of melting of this material. The growth of the share of crystalline regions and linear structures at the introduction of PCL of 70% or more should be accompanied by an increase in the share of straightened chains in the amorphous interlayers, but experimentally, such changes in the compositions have not been recorded. It may be assumed that in the area of phase inversion, the dispersed phase of PLA passes into a dispersion phase, and the radical concentrates mainly in the amorphous structure of PCL due to the rather loose structure of this polymer (molecular mobility in it is almost 30 times higher than in PLA, and the concentration of the radical is ~8 times higher). The radical provides information mainly on the molecular mobility of PCL at its concentration of more than 70%.

Since the melting temperature of PCL lies in the range 50–75 °C, and the glass transition temperature of PLA is in the range 60–75 °C, the radical was introduced into the blend compositions at two temperatures: 50 and 75 °C.

Let us consider the dependences of correlation times on the blend composition when the radical is introduced into the fiber at 75 °C. Correlation times were calculated similarly to the previous experiment. Figure 6b and Figure 7b show the dependences of the characteristic correlation time for the fast and slow components of the EPR spectrum on the blend composition. It can be seen that with the introduction of the radical at 75 °C, the character of the dependences did not change; only higher values of τ were realized in the fibers with a composition ranging from 0 to 50% PCL due to the accessibility of the radical in denser amorphous regions of the material. At a higher content of this polymer in the blend, from 50 to 70%, a kink in the dependence τ on the composition of the composition was also observed. When the concentration of PCL in the blend is higher than 70%, the molecular dynamics in the amorphous regions slows down insignificantly.

The nature of the dependencies of the correlation time on the blend composition when the radical is added at 75 °C differs only by higher values of τ1 and τ2 in the region with high PLA content (up to 50%) than when it is added at 50 °C. At addition of more than 70% PCL the curves coincide irrespective of the temperature of introduction of the radical.

Thus, the patterns of changes in the molecular dynamics in PLA/PCL fiber material are similar to the changes in the enthalpy of melting of this polymer, thus confirming the conclusion about the plasticizing effect of adding PCL up to 50%. At its small concentrations (up to 30%), the distribution of PCL occurs in the form of tiny particles, and perhaps they are also the germs of crystallization. In this case, the possibility for sufficiently straightened macromolecules to adopt the maximally straightened conformation and create additional crystalline and linear structures is realized (as confirmed by DSC data), and, as a result, ΔH τ_1_ and τ_2_ greatly increase. The observed changes can be explained by the transformation of the amorphous structure from a glassy to a highly elastic state. A further increase in the concentration of PCL in the system leads to an increase in the size of the PCL particles (according to the literature data), and as the plasticization effect of the structure of the blend composition decreases, a decrease in the dynamic and structural parameters is observed. In the region of compositions of 50–70% PLA/PCL, a kink is observed in the dependencies of ΔH τ_1_ and τ_2_ on the composition, which is explained by the phase inversion in the blend composition. At a PLA content of 70%, the PCL becomes a continuous phase, the melting enthalpy of both PLA and PCL begins to increase significantly, but the correlation time increases insignificantly, which can be explained by weak changes in the amorphous regions of PCL. Regardless of the temperature of the introduction of the radical into the PLA/PCL system, the character of the change in the correlation time from the blend composition does not change (Figure 6 and Figure 7). When the radical is introduced at 75 °C, τ1 and τ2 are characterized by higher values in comparison with these parameters obtained at 50 °C, only in the region of PCL concentrations less than 70%. At a higher concentration of PCL in the system, regardless of the temperature of the introduction of the radical, these curves coincide.

The fraction of sorbed probe increases significantly with increasing PCL concentration in the material, and in the region of 50–70% PCL, a kink occurs due to the formation of a much softer continuous PCL structure. At a higher addition of this material, the concentration decreases.

Changes in enthalpy of melting, correlations over time, and radical groups in mixtures have similar shapes, and in the regions of 40–70%, a phase transformation takes place, namely the transition of plasma from a dispersed material to a dispersive medium.

**Figure 8:**
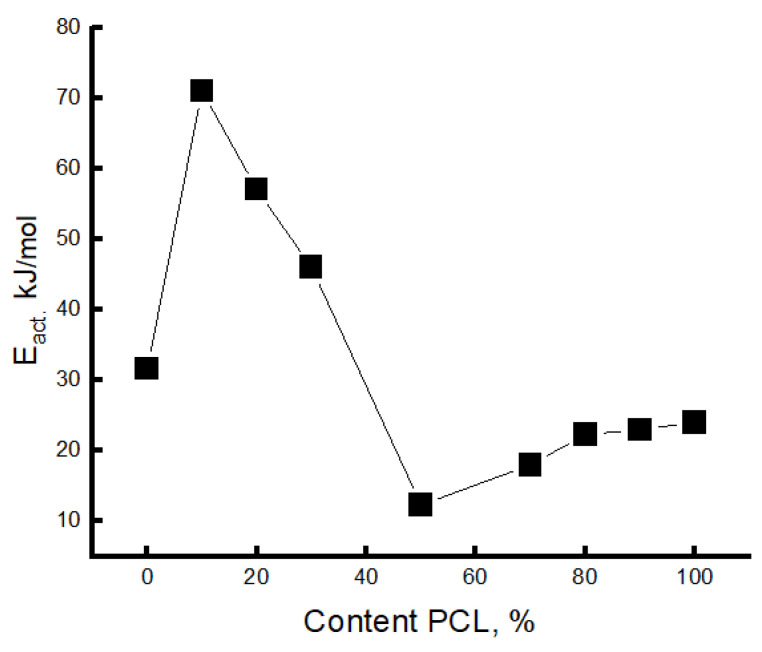
Change in activation energy depending on the composition of the blend.

The effective activation energies Eτ, (Figure 8) were calculated for the fast component when the radical was introduced at 70 °C.

The values of Eτ decrease with increasing PCL concentrations in the system, and the regions of 50/50 and 70/30% concentrations are characterized by the lowest values (the region of phase inversion). At higher PCL concentrations in the system, the Eτ values are slightly increased. The obtained result also confirms the conclusion about the presence of phase inversion in the region of PCL concentration 50–70%, which corresponds to all previous results.

## 4. Conclusions

To evaluate the effect of the blend composition on the structure and segmental mobility, the authors have studied electrospun PLA/PCL fibers at both thermal and dynamic levels through DSC and ESR spectroscopy, correspondingly. The latter characterization was first obtained by the TEMPO-probe ESR technique. Recently, this methodical approach has been specially elaborated for biodegradable polymer blends where the constituents have non-comparable segmental mobility differing by several orders of magnitude.

Comparing thermal findings (melting enthalpy, ΔH) with dynamic characteristics (correlation time, τ,) of PLA/PCL fibers as well as considering the optical microscopy imaging, it was shown that the above characteristic features, depending on the polymer ratio, have three specific areas that correspond to three structural-phase states of the fibers with the proper segmental mobility.

Within the initial 10 wt.% of PCL, a sharp increase in ΔH values and polymer segmental dynamics (τ) was revealed. The trend analysis for both characteristics in combination with microphotographs indicates that the fine dispersion of PCL microparticles in the PLA matrix provides the plasticizing effect of the latter. With increasing the PCL content up to 70%, the values of ΔH and τ have a decreasing trend, indicating the formation of the PLA dispersed phase in dispersive PCL medium. In this case, the PCL plasticizing affecting PLA decreases owing to the reduction in the number of PCL-PLA intersegmental contacts.

As a function of the fiber composition, the changes in the thermal characteristics and segmental mobility have allowed the authors to identify a specific interval of concentration, from 50/50 to 70/30%, where the phase inversion is readily apparent as the transformation of the continuous dispersion medium PLA into its dispersive phase. Here, ΔH and τ have relatively low values, and only starting from 70% of PCL, ΔH significantly increases. In the intercrystalline areas, macromolecular dynamics slows down slightly, since the chain ordering impact of the transitive macromolecules upon segmental mobility must not significantly appear.

In biodegradable PLA/PCL fibers, the observed structural features and segmental mobility alterations should have an essential influence on diffusivity, the profile of controlled release, and mechanical behavior, which is the subject of further investigations designated for biomedical implementation and eco-friendly safety.

## Figures and Tables

**Table 1:** Enthalpy and temperature of melting, cold crystallization, glass transition of nonwoven fiber materials PLA/PCL according to DSC data.

PCL Content in the Blend, %	*ΔH*cr., J/g	*T*melt, °C	*T*gt, °C	*ΔH*cc, J/g	*T*melt, °C	*ΔH*cc/*ΔH*cr.
0	25.7	170.54	63.77	−15.07	86	0.59
10	111	170.1	63.3	−10.7	82.3	0.096
20	83.8	170.1	63	−8.1	80.8	0.096
30	82	170	63	−5	80.1	0.06
50	17.7	169.5	62.7	−3.5	78	0.198
70	3.36	167.9	62			
80	30.6	167.5	61.6			
90	51.7	165.1	61.4			
100	53.84	61.8	61			

## Data Availability

The data presented in this study are available in the article.
